# Transcultural adaptation and validation of the “Hip and Knee” questionnaire into Spanish

**DOI:** 10.1186/1477-7525-12-76

**Published:** 2014-05-17

**Authors:** Enric Castellet, Oscar Ares, Fernando Celaya, Andrés Valentí-Azcárate, Angels Salvador, Ana Torres, Pedro Sesma

**Affiliations:** 1 Orthopedic Department, Hospital Vall d’Hebron, Passeig de la Vall d’Hebrón, 119-129, Barcelona 08035, Spain; 2 Hospital Clinic Barcelona, Barcelona, Spain; 3 Hospital de Sant Pau i Santa Creu, Barcelona, Spain; 4 Clínica Universitaria Pamplona, Pamplona, Spain; 5 Hospital Platón, Barcelona, Spain; 6 Hospital de Cabueñes, Gijón, Spain; 7 Hospital Carlos Haya, Málaga, Spain

**Keywords:** Hip and Knee questionnaire, Translation, Spanish, Validation, Transcultural adaptation

## Abstract

**Background:**

The purpose of the present study is to translate and validate the “Hip and Knee Outcomes Questionnaire”, developed in English, into Spanish. The ‘Hip and Knee Outcomes Questionnaire is a questionnaire planned to evaluate the impact in quality of life of any problem related to the human musculoskeletal system. 10 scientific associations developed it.

**Methods:**

The questionnaire underwent a validated translation/retro-translation process. Patients undergoing primary knee arthroplasty, before and six months postoperative, tested the final version in Spanish. Psychometric properties of feasibility, reliability, validity and sensitivity to change were assessed. Convergent validity with SF-36 and WOMAC questionnaires was evaluated.

**Results:**

316 patients were included. *Feasibility:* a high number of missing items in questions 3, 4 and 5 were observed. The number of patients with a missing item was 171 (51.35%) in the preoperative visit and 139 (44.0%) at the postoperative. *Internal validity:* revision of coefficients in the item-rest correlation recommended removing question 6 during the preoperative visit (coefficient <0.20). *Convergent validity:* coefficients of correlation with WOMAC and SF-36 scales confirm the questionnaire’s validity. *Sensitivity to change:* statistically significant differences were found between the mean scores of the first visit compared to the postoperative.

**Conclusion:**

The proposed translation to Spanish of the ‘Hip and Knee Questionnaire’ is found to be reliable, valid and sensible to changes produced at the clinical practice of patients undergoing primary knee arthroplasty. However, some changes at the completion instructions are recommended.

Level of evidence: Level I. Prognostic study.

## Introduction

Measurement of outcomes has been widely used in orthopedic surgery. During the past decade there has been an increasing number of instruments developed and validated according to the answers offered by patients, whereas previously there were only results based on clinical data provided by the surgeon. Thus, there are increasingly tools available to measure the impact of our procedures in the patients’ health and specific instruments to get to know the result from every anatomic region. Questionnaires are essential tools to measure the impact of a specific surgical technique such as primary knee arthroplasty.

The vast majority of questionnaires have been developed in English. In order to apply them at countries these must be translated and later validated. The process of translation has been previously standardized and it is denominated transcultural adaptation [1-4]. Later on, translated version must be validated in the population of the country of application, showing a reliability, feasibility, validity and sensitivity to change similar to the questionnaire in the original language.

The ‘Hip and Knee Outcomes Questionnaire’ [5] is a questionnaire planned to evaluate the impact in quality of life of any problem related to the human musculoskeletal system. 10 scientific associations linked to disease developed the questionnaire (*American Academy of Orthopedic Surgeons, American Association of Hip and Knee Surgeons, American Orthopedic Society for Sports Medicine, Hip Society, Knee Society, Orthopedic Rehabilitation Association, Orthopedic Trauma Association, Arthroscopy Association of North America, American Orthopedic Foot And Ankle Association, Musculoskeletal Tumor Society).* It consists of 7 items, in which 3 have 4 sub-items, which makes a total of 16 questions with Likert-type answers ranging between 5 or 7 multiple-choices. The “Hip and Knee Outcomes Questionnaire” contains 4 items focused in pain, 2 items in function and 2 items in subject symptoms.

The questionnaire has been applied previously in order to measure functional recovery of patients who had undergone primary hip or knee arthroplasty [6].

In the present study we present the translation process to Spanish of the ‘Hip and Knee Outcomes Questionnaire’ [7] and its subsequent validation of the same as a useful tool for clinical practice in the evaluation of outcomes in primary knee arthroplasty in Spain. We present the analysis of its psychometric properties; feasibility, reliability, validity and sensitivity to change.

## Methods

The cultural adaptation of the questionnaire was carried out at an early stage, developing 1.1 version of the ‘Hip and Knee Outcomes Questionnaire’. The analysis of psychometric properties was performed prospectively with version 1.1 within a group of patients from 12 different hospital centers. Completion of the questionnaires was performed at the time of medical indication of treatment with knee arthroplasty and 6 months after surgery. With this aim, patients filled out versions 1.1 of ‘Hip and Knee Outcomes Questionnaire’ and the already validated Spanish versions of SF-36 and WOMAC questionnaires. These last two were administered for the convergent validity.

The Board of *SEROD* (Spanish Association of Knee) approved the present study. All patients participating in it were informed and signed an informed consent prior to their inclusion in the study.

### Cross-cultural adaptation

The process of cultural adaptation of questionnaires for evaluating results in health follows a methodology that can be summarized as follows:

1. Double translation from the original questionnaire (English) to Spanish. The translation was performed by two translators, an expert in questionnaires as well as an English philologist.

2. Translation revision and composition of the first intermediate version (Spanish).

3. Retro-translation of the first intermediate version in Spanish to the original language (English). A back-translation to English was performed by an independent expertise in questionnaires.

4. Comparison of the retro-translation with the original version and composition of the second intermediate version (Spanish).

5. Composition of the 1.0 version of the questionnaire.

A committee of experts checked version 1.0 of the questionnaire (4 physicians from knee orthopedics) and a committee of 20 patients. During this phase the aim was to evaluate appropriateness, according to its relationship with knee disease and comprehensibility of the items, as well as to assess the final 1.1 version in Spanish.

### Patients

Once we had version 1.1 in Spanish, the psychometric properties were analyzed. 316 patients were included. We analyzed feasibility, validity and reliability within data from visit 1 (before surgery) and secondly from visit 2 (6 months postoperative). Inclusion criteria for the present study were 1) Patients undergoing primary total knee arthroplasty, 2) Signed informed consent had been obtained from the patient, 3) The patient was at least eighteen years old on the day of signing the informed consent and 4) The patient was cognitively intact, fluent in Spanish, and sufficiently literate to complete the self-administered questionnaire. Exclusion criteria were patients who the investigator believed that might fail to comply with the protocol.

Next we described scorings registered from the patients during visits 1 and 2, comparing these scorings from the studied questionnaire to figure out if there were significant differences between the moments of measurement and to analyze the sensitivity to change from the questionnaires used in the present study.

### Statistical analysis

The statistical analysis was performed at both evaluation-times (before and after surgery). The analyzed properties in each case were the following:

a. *Feasibility:* this was evaluated according to the percentage of no-answer in every item (missing items) and to the percentage of patients that did not answer some item, as well as to the completion time of the studied questionnaires. Moreover, ceiling effect was studied (percentage of patients with maximum score, indicating a better clinical situation), together with floor effect (percentage of patients with minimum score, indicating a worse clinical outcome), for every item and for global scoring of every instrument used.

b. *Validity*: internal validity was evaluated by means of the analysis of item-rest correlation and Exploratory Factor Analysis (EFA) of the answers to the questionnaire’s items. The item-rest correlation allows us to know the power of discrimination from every item related to the scale’s global score. Values lower than 0.2 would advise the removal of that item due to its low power of discrimination. Regarding EFA, the following were obtained:

Kaiser-Meyer-Olkin: measures the sampling adequacy, which should be greater than 0.5 for a satisfactory factor analysis to proceed. It is larger when the partial correlations among variables are small [8].

- Bartlett’s test of sphericity [9]: used to test the null hypothesis that the variables in the population correlation matrix are uncorrelated. If we try to get together variables from a questionnaire as factors, there must be certain relationship between the variables. Consequently, if we reject the null hypothesis of Bartlett’s test, the factor analysis makes sense.

- Following the extraction method by Kaiser, we present all the elements with initial autovalues higher than 1. Nevertheless, considering the validation of the original version, which considers that there is a unidimensional structure of the ‘Hip and Knee Questionnaire’, we will present the variance percentage that explains the factor with more weight and the correlation for every item within that only factor that would explain a sufficient minimum percentage.

- Convergent validity: correlation analysis (Spearman’s Rho) between the scores of the previous studied instrument and two questionnaires already translated and validated in our country; SF-36 and WOMAC.

c. *Reliability:* internal consistence was contrasted through Cronbach’s alpha [10].

d. *Sensitivity to change:* analysis of the differences within the mean scores between before and after surgery (*t*-test for related samples and Wilcoxon signed-ranked test). Furthermore, we measured the ‘Effect Size’ (Cohen’s *d*) [11] in order to know the effect size in each case after surgery.

## Results

### Translation results

During the first stage of translation, the only item that seemed to have problems of feasibility, demonstrating comprehensive problems, was item 1 *“Durante la semana pasada ¿ha notado su cadera/rodilla* ***entumecida****?” (During the past week, how stiff was your hip/knee?)*; which was not answered by 4 out of 10 interviewed patients. This item 1 had low comprehensibility (1.79) and relevance slightly lower than 3.

The panel of experts later confirmed this fact, which made it advisable to change the composition of the item, especially taking into account that 4 out of 10 interviewed patients did not answer. Several options were discussed in order to change the composition (Tense, Rigid, Numb, Feeling of rigidness, Painful).

Finally, the definitive version of question 1 was:

1. *Durante la semana pasada ¿ha notado su cadera/rodilla* ***agarrotada****? (During the past week, how stiff was your hip/knee?)*

The final version of the questionnaire was version 1.1 (Additional file 1). Once obtained the Spanish version 1.1, psychometric properties were analyzed.

### Preoperative results

Data from 333 patients was analyzed.

a) *Feasibility:*

Missing items (percentage of no-answer): Hip and Knee Questionnaire; a high number of missing items was observed in questions 3, 4 and 5; which present multiple choice of answers for each studied joint (Table 1). The amount of missing items at the SF-36 questionnaire was very low, between 6 (1.8%) and 14 (4.2%), as well as in the WOMAC form, varying from 9 (2.7%) to 16 (4.8%); always under 5% in both questionnaires.

Percentage of patients with missing items: 171 patients (51.35%) had at least one missing answer in the Hip and Knee Questionnaire. On the other hand, the SF-36 presented only 31 patients (9.33%) with one or more missing items and 35 patients (10.51%) for the WOMAC questionnaire.

Ceiling and floor effects: both effects were almost invaluable for the Hip and Knee Questionnaire, and very low for WOMAC (pain, stiffness and physical function). As for SF-36 health questionnaire, the ceiling and floor effects were null for the two summary scores, as these were standardized with the mean of the Spanish general population. Regarding the gross dimensions (scores 0–100), the floor effect (worst possible score) was small, always less than 10%; as well as the ceiling effect (best possible score), which was also low except for the Role Emotional dimension.

b) *Internal validity:* exploratory factor analysis (EFA) of the Hip and Knee reveals the presence of more than one dimension, although the first factor explains 32% of the variance, which speaks in favor of the existence of a latent unidimensional structure. The item-rest correlation advises the removal of item 6 (*Which of the following statements best describes your ability to get around most of the time during the past week?*), of which coefficient is less than 0.20.

c) *Convergent validity:* correlation coefficients of the Hip and Knee Questionnaire compared to the WOMAC scale are moderate, as it had been hypothesized, which speaks in favor of the adequate validation of the studied questionnaire. Correlations of the Hip and Knee with both WOMAC pain and functional were 0.641, and 0.533 when compared to WOMAC-stiffness. All coefficients were significant at level 0.01 (bilateral). When comparing Hip and Knee with SF-36, correlation coefficients are also moderate, however, these are more proximal to 0.4 than to 0.7. This correlation also affirms the correct validation of the studied questionnaire (Table 2).

d) *Reliability:* Cronbach’s alpha was 0.864.

**Table 1:** Missing items for every item of the ‘Hip and Knee Outcomes Questionnaire’ during the preoperative visit

	**Number of missed**	**% of missed**
1. During the past week, how tense was your hip/knee?	21	6,3%
2. During the past week, how swollen was your hip/knee?	18	5,4%
3. Walking on flat surfaces.		
a. Right hip	139	41,7%
b. Left hip	145	43,5%
c. Right knee	65	19,5%
d. Left knee	72	21,6%
4. Going up or down stairs		
a. Right hip	149	44,7%
b. Left hip	151	45,3%
c. Right knee	70	21,0%
d. Left knee	77	23,1%
5. Lying in bed at night		
a. Right hip	142	42,6%
b. Left hip	149	44,7%
c. Right knee	68	20,4%
d. Left knee	65	19,5%
6. Which of the following statements best describes your ability to get around most of the time during the past week?	16	4,8%
7. How difficult was it for you to put on or take off socks/stockings during the past week?	15	4,5%

**Table 2:** Correlations Hip and Knee – SF-36 visit 1

	**SF-36 physical component**	**SF-36 mental component**	**SF-36 physical function**	**SF-36 role physical**	**SF-36 pain**	**SF-36 general health**	**SF-36 vitality**	**SF-36 social function**	**SF-35 role emotional**	**SF-36 mental health**
Hip and Knee Global Scoring	.457	.360	.458	.480	.537	.316	.371	.405	.406	.333

Spearman’s correlation is significant at level 0.01 (bilateral).

### Postoperative results (6 months after surgery)

Data from 316 patients was analyzed.

a) *Reliability:* Cronbach’s alpha was 0.849.

### Sensitivity to change

As we can see in Table 3, there are statistically significant differences in all mean scorings when comparing before and after surgery (*t*-test for related measurements and Wilcoxon signed-ranked test). In conclusion, there seems to be a significant improvement after undergoing joint replacement.

**Table 3:** Sensitivity to change of the questionnaires: mean differences between scorings at visits 1 and 2

**Sales and measurement instruments**	**N**	**Mean**	**Standard error of the mean**	**Median**	**Standard deviation**	**Minimum**	**Maximum**	**Size effect**
Hip and Knee v1 global score	299	14.39	0.34	14.17	5.95	0	31.5	1.409
Hip and Knee v2 global score	281	6.00	0.29	4.83	4.91	0	29.25	
SF-36 mental component v1	324	44.21	0.67	43.65	12.02	13.58	68.66	0.297
SF-36 mental component v2	301	47.78	0.64	50.35	11.12	10.00	69.05	
SF-36 physical component v1	324	31.84	0.41	30.53	7.43	14.97	53.72	1.078
SF-36 physical component v2	301	39.85	0.50	40.44	8.67	14.97	60.54	
WOMAC pain v1	322	15.88	0.23	16	4.10	5	25	1.541
WOMAC pain v2	305	9.55	0.21	9	3.69	5	25	
WOMAC functional v1	314	56.94	0.75	56	13.23	18	85	1.579
WOMAC functional v2	301	36.05	0.77	35	13.44	17	82	
WOMAC stiffness v1	329	6.40	0.11	6	2.03	2	10	1.187
WOMAC stiffness v2	307	3.98	0.10	4	1.79	2	10	

p value was statistically significant in all levels (p < 0.001).

Finally, in order to elucidate the size effect, Cohen’s *d* was measured considering results lower than 0.2 as small size; moderate when close to 0.5 and big when results were greater than 0.8. The only effect small in magnitude was for the mental component of SF-36. Instead, within the rest of scales, this effect was high, which contributes with more evidence of the correct validation of the scale that we are studying (in this case, in terms of sensitivity to change).

## Discussion

Due to the internationalization of surgical techniques and to the increasing number of multinational research projects, it is necessary to adopt our measuring instruments of results to other countries other than the instrument’s original. The vast majority of these instruments have been designed in English. Therefore, it was necessary to establish requisites for the translation process, and these were applied in the present study [4]. However, the term *cross-cultural adaptation* refers to a wider process that includes not only translation but also the cultural adaptation to the new country of application. Different habits of life within different cultures can alter the results of a questionnaire. As an example, during the adaptation of the IKDC test to its Brazilian version the term “skiing” was substituted by “surfing” [12].

The present study has centered in the ‘Hip and Knee Outcomes Questionnaire’. This questionnaire was developed originally outside Spain. The result of the cultural adaptation was version 1.1 of the questionnaire.

During the translation process it was observed that the only item with feasibility problems, showing comprehensibility problems, was item 1 “*Durante la semana pasada ¿ha notado su cadera/rodilla* ***entumecida****?”*, which was not answered by 4 out of 10 interviewed patients.

This item 1 had a low comprehensibility, and an appropriateness slightly lower than 3, probably because the question was not understandable. The panel of experts confirmed this fact, especially since this question was not answered by 4 out of 10 patients and advised changing the composition of the item. Several alternative options were proposed, and the final composition was that exposed in version 1.1: *1. Durante la semana pasada ¿ha notado su cadera/rodilla agarrotada?*

Once we achieved a final cultural-adapted version of the ‘Hip and Knee Questionnaire’, a multicenter and prospective study was conducted, in which we collected patient data that were tested before and after primary knee arthroplasty. This makes a large group of patients from different hospital centers around Spain, avoiding geographic bias. This evaluation test was implemented not only for the mentioned questionnaire, but for two validated scale; a specific one (WOMAC) and a generic (SF-36), widely used in our country. The *SF-36 Health Questionnaire,*[13,14] measures health-related quality of life, applicable to any group of population. Its Spanish version has already been validated [15-17]. The *WOMAC* questionnaire [18] is a specific instrument developed to evaluate the impact in quality of life of osteoarthritis. The questionnaire has been previously translated and validated in Spain [19]. With these data, we proceeded to the analysis of psychometric properties of the ‘Hip and Knee’: feasibility, reliability, validity and sensitivity to change.

Regarding the *feasibility* of the scales, the Hip and Knee questionnaire presented, during both visits, a high percentage of missing items at questions 3, 4 and 5. These questions refer to multiple-choice answers for every joint at study, and a possible cause of failure could be the instructions not being clear enough: the patient has to mark and answer for every line (a, b, c, d), and not exclusively mark the line that refers to the operated joint in each case. They understood that they were asking only for the affected knee joint when in fact asked about the status of both knees and hips. We believe that the questionnaire is still appropiate for the following reasons: first, the patients answered the question correctly on their affected joint; secondly, despite some missing items the questionnaire responses have shown its feasibility, internal validity, reliability and sensitivity to change. In this sense, it would be recommended having the doctor revising its correct complementation.

These difficulties were reflected in the high percentage of patients with at least one missing item in the Hip and Knee within both visits (51.33% at visit 1, 44.0% at visit 2). Regarding WOMAC and SF-36 questionnaires, these percentages were clearly lower.

The analysis of the *ceiling and floor effect* reflected scorings almost insignificant.

With regard to the *internal validity*, the EFA carried out with data from the two evaluation moments of the study, allowed verifying the existence, just at it is stated in the original version, of a dimension that explained a higher percentage of variance (32% approximately), together with an acceptable internal consistence (Cronbach’s alpha 0.864 and 0.849, respectively). Except for item 6 (*Which of the following statements best describes your ability to get around most of the time during the past week?*) during visit 1, the item-rest correlation was always greater than 0.2, and even 0.3. Results from this item at the preoperative phase would advise its removal. However, at the postoperative visit, the results from this item were satisfactory. A possible explanation could be a greater involvement of the surgeon when answering the questionnaire 6 months after surgery.

Regarding the *convergent validity*, a significant association with the specific instrument (WOMAC) was found (moderate-high), supporting the correct validation of the questionnaire. In the same line, when compared to the generic scale SF-36, the associations were found to be significant, in a higher intensity when compared to the functional dimensions of SF-36 rather than to Role Emotional or Social, just as we expected.

Finally, with regards to the *sensitivity to change* of the tests, we found, in all cases, statistically significant differences between mean scores at visit 1 compared to visit 2. We observed, in all cases, an improvement after the surgical procedure, reinforcing once more, the correct validation of the questionnaires to find changes in quality of life of patients following a knee procedure.

Several limitations should be taken into consideration when reviewing the present manuscript. Firstly, no patients with severe hip dysfunction were included. Second, test-retest reliability, and thus, correlation coefficient, were not provided, even though it has been already described in previous translations.

In conclusion, from the analysis performed of the ‘Hip and Knee Outcomes Questionnaire’, it is proved that this scale is reliable, valid and sensible to the produced changes in the clinical framework of patients undergoing primary knee arthroplasty. We recommend having the doctor revising its correct complementation.

Moreover, its limited extension makes it possible to be included in the regular clinical practice of medical doctors.

## Competing interests

Each author certifies that he or she has no commercial associations (e.g., consultancies, stock ownership, equity interest, patent/licensing arrangements, etc.) that might pose a conflict of interest in connection with the submitted article. No benefits in any form have been received or will be received from a commercial party related directly or indirectly to the subject of this article.

## Authors’ contributions

EC participated in acquisition of patients’ data, revised the manuscript, contributed to the conception and design of the study and supervised the statistical analysis. OA participated in drafting the article, carried out the statistical analysis and designed the manuscript. FC participated in acquisition of patients’ data, analysis of data and supervision of the manuscript. AVA participated in acquisition of patients’ data, interpretation of results and supervision of the manuscript. AS participated in acquisition of patients’ data, contributed to conception and design and revision of results. AT participated in acquisition of patients’ data, analysis and interpretation of statistical analysis. PS participated in revision of the manuscript and interpretation of results. All authors read and approved the final version of the manuscript.

## Supplementary Material

Additional file 1 Hip and knee questionnaire. Click here for file
