# Supplementary material for: Transcultural adaptation and validation of the “Hip and Knee” questionnaire into Spanish
Source: Health Qual Life Outcomes. 2014 May 17;12:76. doi: 10.1186/1477-7525-12-76 (PMC4035858; doi:10.1186/1477-7525-12-76)
Supplement: Additional file 1 — Hip and knee questionnaire. [file 1477-7525-12-76-S1.doc]

**HIP AND KNEE QUESTIONNAIRE**

**Instrucciones**

*Por favor, responda a las siguientes preguntas en relación a la cadera / rodilla de la que está siendo tratado o que está bajo seguimiento. Si se tratara de AMBAS caderas / rodillas, responda pensando en la que* ***más le molesta****. Todas las preguntas se refieren a cómo se ha sentido, en términos generales, durante la* ***semana pasada****. Si está recibiendo tratamiento por una lesión que se produjo hace menos de una semana, responda basándose en el tiempo que ha transcurrido desde entonces.*

**1.** Durante **la semana pasada** ¿ha notado su cadera/rodilla **agarrotada**? (Rodee una sola respuesta con un círculo)

1) Nada 2) Algo 3) Bastante 4) Mucho 5) Muchísimo

**2.** Durante **la semana pasada** ¿tuvo la cadera/rodilla **hinchada**? (Rodee una respuesta)

1) Nada 2) Algo 3) Bastante 4) Mucho 5) Muchísimo

***Las instrucciones que se presentan a continuación hacen referencia a las preguntas 3, 4 y 5.***

Pensando en la **semana pasada**, díganos cuánto le dolieron las caderas / rodillas al realizar estas actividades. (Rodee UNA SOLA respuesta por línea, la que mejor describa la capacidad media de cada articulación).

|  | **Nada doloroso** | **Algo doloroso** | **Bastante doloroso** | **Muy doloroso** | **Extremadamente doloroso** | **No podía debido al dolor en la cadera / rodilla** | **No podía por otras razones** |
| --- | --- | --- | --- | --- | --- | --- | --- |
| **3.** Caminar por una superficie **llana** |  | | | | | | |
| Cadera derecha | 1 | 2 | 3 | 4 | 5 | 6 | 7 |
| Cadera izquierda | 1 | 2 | 3 | 4 | 5 | 6 | 7 |
| Rodilla derecha | 1 | 2 | 3 | 4 | 5 | 6 | 7 |
| Rodilla izquierda | 1 | 2 | 3 | 4 | 5 | 6 | 7 |

|  | **Nada doloroso** | **Algo doloroso** | **Bastante doloroso** | **Muy doloroso** | **Extremadamente doloroso** | **No podía debido al dolor en la cadera / rodilla** | **No podía por otras razones** |
| --- | --- | --- | --- | --- | --- | --- | --- |
| **4.** Subir o bajar escaleras |  | | | | | | |
| Cadera derecha | 1 | 2 | 3 | 4 | 5 | 6 | 7 |
| Cadera izquierda | 1 | 2 | 3 | 4 | 5 | 6 | 7 |
| Rodilla derecha | 1 | 2 | 3 | 4 | 5 | 6 | 7 |
| Rodilla izquierda | 1 | 2 | 3 | 4 | 5 | 6 | 7 |

|  | **Nada doloroso** | **Algo doloroso** | **Bastante doloroso** | **Muy doloroso** | **Extremadamente doloroso** | **No podía debido al dolor en la cadera / rodilla** | **No podía por otras razones** |
| --- | --- | --- | --- | --- | --- | --- | --- |
| **5.** Permanecer tumbado en la cama por la noche |  | | | | | | |
| Cadera derecha | 1 | 2 | 3 | 4 | 5 | 6 | 7 |
| Cadera izquierda | 1 | 2 | 3 | 4 | 5 | 6 | 7 |
| Rodilla derecha | 1 | 2 | 3 | 4 | 5 | 6 | 7 |
| Rodilla izquierda | 1 | 2 | 3 | 4 | 5 | 6 | 7 |

**6.** ¿Cuál de las siguientes afirmaciones describe **mejor** su capacidad de movimiento durante la mayor parte del tiempo de la **semana pasada**? (Rodee una respuesta).

| 1 | No necesité ayuda de ningún tipo ni nada sobre lo que apoyarme. |
| --- | --- |
| 2 | La mayor parte del tiempo caminé sin ayuda ni apoyos. |
| 3 | La mayor parte del tiempo utilicé un bastón o una muleta para moverme. |
| 4 | La mayor parte del tiempo utilicé dos bastones, dos muletas o un andador para moverme. |
| 5 | Utilicé una silla de ruedas. |
| 6 | La mayor parte del tiempo utilicé otros apoyos o alguien me ayudó a moverme. |
| 7 | Fui incapaz de moverme. |

**7.** ¿Le resultó difícil ponerse o quitarse los calcetines / las medias durante la **semana pasada**? (Rodee una respuesta)

1) Nada difícil 2) Algo difícil 3)Bastante difícil 4) Muy difícil 5) Extremadamente difícil 6) No pude hacerlo
